# Bead Array Direct rRNA Capture Assay (rCapA) for Amplification Free Speciation of *Mycobacterium* Cultures

**DOI:** 10.1371/journal.pone.0032575

**Published:** 2012-03-02

**Authors:** Hans de Ronde, Paula González Alonso, Dick van Soolingen, Paul R. Klatser, Richard M. Anthony

**Affiliations:** 1 KIT Biomedical Research, Royal Tropical Institute, Amsterdam, The Netherlands; 2 Department of Molecular Cell Biology, Faculty of Earth and Life Sciences, VU University Amsterdam, Amsterdam, The Netherlands; 3 Tuberculosis Reference Laboratory, Centre for Infectious Disease Control, National Institute for Public Health and the Environment (RIVM), Bilthoven, The Netherlands; McGill University, Canada

## Abstract

*Mycobacterium* cultures, from patients suspected of tuberculosis or nontuberculous mycobacteria (NTM) infection, need to be identified. It is most critical to identify cultures belonging to the *Mycobacterium tuberculosis* complex, but also important to recognize clinically irrelevant or important NTM to allow appropriate patient management. Identification of *M. tuberculosis* can be achieved by a simple and cheap lateral flow assay, but identification of other *Mycobacterium* spp. generally requires more complex molecular methods. Here we demonstrate that a paramagnetic liquid bead array method can be used to capture mycobacterial rRNA in crude lysates of positive cultures and use a robust reader to identify the species in a direct and sensitive manner. We developed an array composed of paramagnetic beads coupled to oligonucleotides to capture 16 rRNA from eight specific *Mycobacterium* species and a single secondary biotinilated reporter probe to allow the captured rRNA to be detected. A ninth less specific bead and its associated reporter probe, designed to capture 23S rRNA from mycobacteria and related genera, is included as an internal control to confirm the presence of bacterial rRNA from a GC rich Gram variable genera. Using this rRNA capture assay (rCapA) with the array developed we were already able to confirm the presence of members of the *M. tuberculosis* complex and to discriminate a range of NTM species. This approach is not based on DNA amplification and therefore does not require precautions to avoid amplicon contamination. Moreover, the new generation of stable and cost effective liquid bead readers provides the necessary multiplexing potential to develop a robust and highly discriminatory assay.

## Introduction

Automated liquid culture is faster than culture on solid media and in fact the gold standard in the diagnosis of tuberculosis (TB). For these reasons liquid culture is widely used to detect and confirm infection with *Mycobacterium tuberculosis*. Critically liquid culture is also more effective at recovering other members of the *Mycobacterium* genus [1], [2]. These non *M. tuberculosis* complex species, the so called non tuberculous mycobacteria (NTM), when cultured may represent environmental contaminants, colonization or, particularly in immunosuppressed or aging populations, represent genuine pathogens [3], [4]. Not only in the Western world, but also in Africa the clinical importance of NTM disease seems highly underestimated, especially in settings where HIV is prevalent [5]. This increasing recovery of NTM with modern culture methods, as well as a likely increase in their importance in countries with a low burden of tuberculosis and possibly throughout the world [6], makes *Mycobacterium* species identification from clinical cultures increasingly important. Also, the treatment for *M. tuberculosis* complex infection requires at least 6 months and is specifically tailored to this organism. Disease caused by other (NTM) species requires different drug combinations [3] and thus it is necessary to confirm the species of mycobacteria cultured in order to provide appropriate therapy.

Currently, speciation of cultured mycobacterial species is most effectively achieved by either a simple lateral flow test [7], which in fact only confirms the presence of a member of the *M. tuberculosis* complex. Alternatively, mycobacterial DNA amplification may be used, followed by a “macro array” hybridization assay, which can specifically identify a wide range of *Mycobacterium* species [8]. Although this method combines a high sensitivity and specificity, it requires an infrastructure for molecular assays and the tests are expensive and have a turn-around-time of several hours. Another system available for mycobacterial culture identification is the Gen-Probe AccuProbe method which uses species specific probes and a dedicated reader to identify cultures. This method is effective, but only a single probe can be tested in each assay and if negative the next most likely target must be tested increasing the cost of the identification [9]. Therefore, the popularity of this method is decreasing.

Here we propose an alternative concept with the potential to be simpler than an DNA amplification/macro array approach, but like the AccuProbe method not susceptible to the risk of PCR contamination and associated requirements for amplicon clean and exposed rooms, while providing much more information on species identification than a lateral flow assay [10], [11]. To achieve this, we first inactivate and mechanically lyse an aliquot of a positive liquid culture in a buffer, which both denatures the protein present and stabilizes the mycobacterial RNA. This crude extract is then hybridized, in the RNA stabilizing buffer, to a bead array of eight paramagnetic xMAP Luminex beads (Liminex Corp., Texas, USA) coupled to oligonucleotides designed to capture 16S rRNA from specific *Mycobacterium* species and a secondary biotinilated reporter probe. A ninth bead, designed to non-specifically capture 23S rRNA from mycobacteria and related genera and its associated reporter probe, is also included as an internal control to confirm the presence of bacterial rRNA from a GC rich Gram variable genus. After hybridization the beads and captured RNA are magnetically concentrated, the lysis buffer is removed, replaced with analysis buffer and the signal developed. The processed beads are subsequently analysed using a MAGPIX™ (Luminex Corp., Texas, USA) device and the hybridization profile used to identify the species present in the culture. The MAGPIX platform, which unlike previous Luminex readers is based on a CCD camera and LED illuminated flow cell rather that a flow cytometer, is a compact robust devise capable of identifying and quantifying the signal collected on an suspension of up to 50 different bead species. Beads can either be purchased pre coupled to assay reagents or, as in this report, coupled by the end user to develop novel assays.

The *Mycobacterium* genus consists of more than 100 recognized species. So in order to demonstrate the concept and develop and optimize protocols presented in this paper we developed a pilot test bead array to identify nine species. None the less this method successfully discriminated a useful range of species previously recovered from patient material and associated with different clinical significance [3]. This array could be easily extended with the inclusion of additional beads for other species. In combination with a newly available, robust reader for bead arrays we feel the rCapA presented could be particularly suitable for use in diagnostic mycobacteriology. The general approach of this assay could be applied in many other applications, such as, the direct capture of rRNA from other culture samples where speciation is required [12], rRNA capture from heavily contaminated material such as soil [10] or the direct capture of mRNA for expression analysis [11].

## Methods

### Strains used

We used a collection of strains available in our laboratory [11], [13], [14], [15] for developing and testing the methodology (Table 1). Cultures were grown at 30°C for *M. marinum*, or 36°C for other *Mycobacterium* species to early log phase in 10 mL of Middlebrook 7H9 liquid media supplemented with OADC. Subsequently, we tested a series of 14 Mycobacteria Growth Indicator (MGIT) Tubes (BD NJ, USA), one uninoculated, cultured at the RIVM that were previously identified using reverse line blot tests (Hain Lifescience Nehren, Germany) for identification, which we used to check the suitability of our method for use in combination with the MGIT culture system (Table 2). Species other than mycobacteria were grown in nutrient broth in a shaking incubator, 200 rpm, overnight at 37°C.

**Table 1:** Middlebrook 7H9 cultures used in this study.

Strain	Species	Source (, reference)
Mtb1A	*M. tuberculosis*	RIVM myc5414
B15	*M. tuberculosis*	Resistant mutant of Mtb72, 13
R181	*M. tuberculosis*	Resistant mutant of Mtb72
Mtb72	*M. tuberculosis*	KIT subculture of ATCC35801
BCG_1	*M. bovis*	KIT sub culture of ATCC35733
*M.afri*	*M. africarnum*	RIVM myc5544
H15	*M. tuberculosis*	Resistant mutant of Mtb72, 15
H103	*M. tuberculosis*	Resistant mutant of Mtb72, 15
R190	*M. tuberculosis*	Resistant mutant of Mtb72
RB16	*M. tuberculosis*	Resistant mutant of Mtb72
RB19	*M. tuberculosis*	Resistant mutant of Mtb72
M mic1	*M. microti*	KIT collection
M avi1a	*M. avium*	KIT collection, 14
M avi15	*M. malmoense* *	KIT collection
M avi30	*M. avium*	RIVM 13528-1071
M avi60	*M. avium*	RIVM 6450-204
M xen6	*M. xenopi*	KIT collection, 14
M abs0278	*M. abscessus*	RIVM 1011100278
M abs2283	*M. abscessus*	RIVM 1011002328
M kan10	*M. kansasii*	KIT collection
M kan20	*M. kansasii*	KIT collection, 14
M scr1	*M. scrofulacum*	KIT collection, 14
M scr2	*M. scrofulacum*	KIT collection
M malm1	*M. malmoense*	KIT collection
M malm2328	*M. malmoense*	RIVM 1011002328
M malm0007	*M. malmoense*	RIVM 1011100007
M gor19	*M. gordonae* *	RIVM C809
Staph	*Staphylococcus aureus*	Clinical isolate 3341-7, 11
E. coli	*Eschericia coli*	KIT 4069-2
*N ast1*	*Nocadia asteroids* *	KIT collection isolated from human sputum
*N ast2*	*Gordonia bronchialis* *	KIT collection isolated from human sputum

KIT = Royal Tropical Institute, The Netherlands.

RIVM = Dutch Centre for Infectious Disease Control.

* = Species identification based on sequencing a portion of the 16S rRNA.

**Table 2:** MGIT cultures used.

Culture ID	Identification by GenoType Assay
Mtb 887	*M. tuberculosis*
Mtb 825	*M. tuberculosis*
M avi 569	*M. avium*
M avi 863	*M. avium*
M xen 346	*M. xenopi*
M abs 720	*M. abscessus*
M abs 742	*M. abscessus*
M kan 793	*M. kansasii*
M kan 884	*M. kansasii*
M mal 682	*M. malmoense*
M mar 674	*M. marinum*
M gor 587	*M. gordonae*
M gor 849	*M. gordonae*

### Selection of initial set of probes

**Figure 1:**
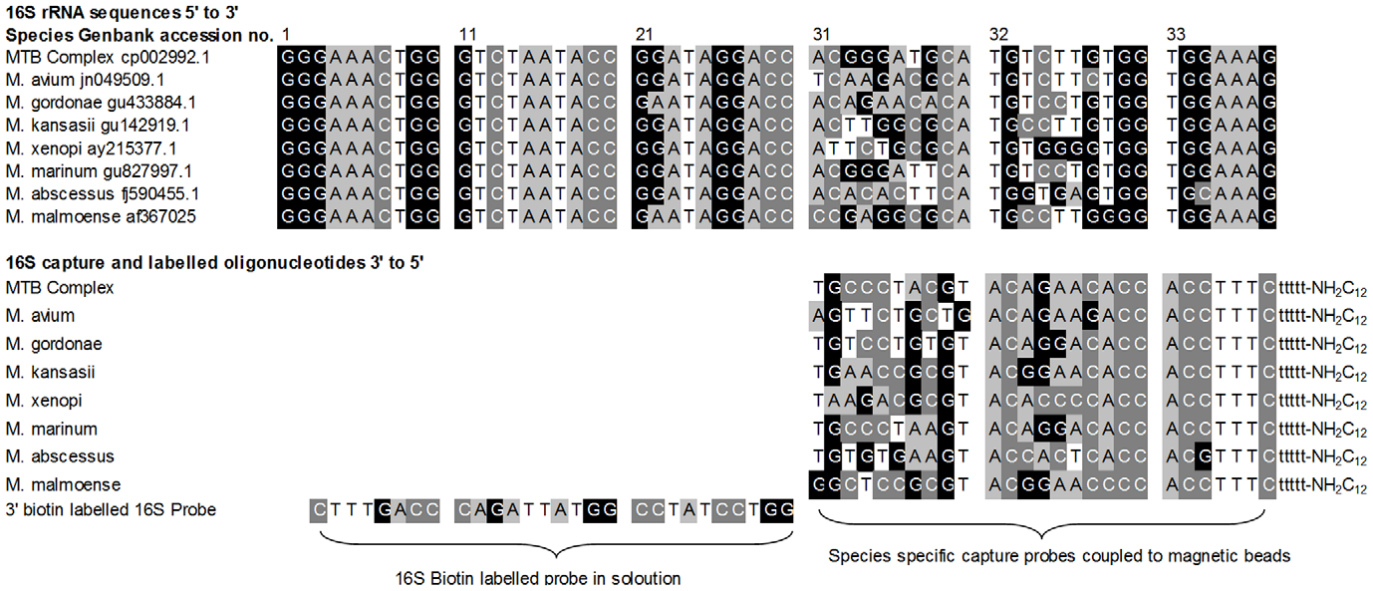
Schematic alignment of the bead array and target rRNA assembly.

The probes selected are listed in Table 3 and the rCapA and predicted assembly of the probes, in the presence of selected mycobacterial rRNAs, is illustrated in Figure 1. The species specific probes tested are based on a variable genomic 16S rRNA region previously shown to be suitable for mycobacterial speciation [14]. Specifically, the mycobacterial probe used to identify the MTB complex in this study is closely related to a probe, pTub1, previously validated for use in a reverse hybridization assay [14]. The associated 16S biotin labelled reporter probe was selected to bind to the rRNA directly 5′ of the capture probe, as this arrangement has been shown to result in the highest positive signal [10], [11].

**Table 3:** Probes used in this study.

Capture probes	Coupled to MagPlex bead number	5′ amine modification	GC (%)	BASES
**16S rRNA**				
M tuberculosis	27	5′-NH_2__C_12_-tttttCTTTCCACCACAAGACATGCATCCCGT	43.8	32
M avium	14	5′-NH_2__C_12_-tttttCTTTCCACCAGAAGACATGCGTCTTGA	43.8	32
M gordonae	72	5′-NH_2__C_12_-tttttCTTTCCACCACAGGACATGTGTCCTGT	43.8	32
M kansasii	39	5′-NH_2__C_12_-tttttCTTTCCACCACAAGGCATGCGCCAAGT	46.9	32
M xenopi	28	5′-NH_2__C_12_-tttttCTTTCCACCACCCCACATGCGCAGAAT	46.9	32
M marinum	65	5′-NH_2__C_12_-tttttCTTTCCACCACAGGACATGAATCCCGT	43.8	32
M abscessus	34	5′-NH_2__C_12_-tttttCTTTGCACCACTCACCATGAAGTGTGT	40.6	32
M malmoense	44	5′-NH_2__C_12_-tttttCTTTCCACCCCAAGGCATGCGCCTCGG	56.3	32
**23S rRNA**	-		-	-
23S+Control	26	5′-NH_2__C_12_-tttttCCCTATTCAGACTCGCTTTCGCTGCGGCTA	48.6	35
**Detector probes**		**3′ Biotin modification**		
16S Detector	In solution	GGTCCTATCCGGTATTAGACCCAGTTTC-3′-Biotin	50	28
23S Detector	In solution	CCCCACCCGGGTTAACCTCGCGACATG-3′-Biotin	66.7	27

Probes selected to target mycobacteria of other species were selected to lie directly 5′ of the MTB complex reporter probe binding to a hyper-variable region of the 16S rRNA (Figure 1). This allowed the same reporter probe to be used for all bacterial species targeted.

In contrast, the 23S capture and reporter probe were selected so as to bind to a wide range of microorganisms under the hybridization conditions selected. This probe pair was designed to act as a positive control, to indicate the presence and approximate concentration of any rRNA present from the *Mycobacterium* or closely related genera.

**The liquid bead array** was prepared by covalently coupling each 5′ amine modified oligonucleotides to a 5′12 carbon spacer (Table 3) and five thymine residues [16] to 1×10^6^ unique microspheres as described by the manufacturer (carbodiimide coupling of amine modified oligonucleotides to MagPlex™-C magnetic carboxilated xMAP® microspheres, Luminex Corp.). Once prepared coupled microspheres were re-suspended in 200 ul of 1×TE buffer pH 8 and stored at 4°C in the dark until needed. These solutions of labeled beads were then combined to produce bead mixtures (liquid arrays) and suspended in 2×SSPE (0.3 M NaCl, 0.02 M NaH2PO4, 0.002 M EDTA pH 7.4) as required to a final concentration 45,000 of each bead species per ml. Aliquots of these mixtures were used in the assay with approximately 1,500 beads of each species used per reaction.

### Bacterial killing and lysis

Mycobacterial cultures were heated to 80°C for 30 min prior to lysis. Bacteria (0.5 ml for Middlebrook culture and 4 ml for MGIT cultures) were then pelleted by spinning at 10,000 g for 5 min in a micro-centrifuge and the supernatant discarded. The pelleted cells were then re-suspended, at 1 to 2× original concentration for Middlebrook cultures and 20× concentration for MGIT cultures, in 5 M GTC buffer (5 M guanidine thiocyanate, 0.05 M Tris, 0.02 M EDTA pH 8, 1% triton X-100). Then to 150 ul aliquots of the concentrated cells 0.2 g of 0.1 mm diameter Zirconium/Silica beads (BioSpec products, Inc.) was added and the cells mechanically lysed by shaking in a Retch shaker (Retsch, Düsseldorf, Germany) at max speed (30 Hz) typically for 10 min but up to 60 min to investigate the effect of increased shaking.

### Hybridisation reactions

To 100 µl aliquots of the bacterial lysate prepared above 5 µl of a 5 µM solution of each labeled detector ologonucleotide and 33 µl of a bead mixture (45,000 of each bead species/ml) was added. The mixture was then heated to 95°C for 3 min and held at 50°C with gentle shaking to allow the hybridization to proceed. For method development hybridization was allowed to continue overnight, but hybridization kinetics were studied by removing aliquots of selected samples at 1, 3, 5, and 18 hrs. After hybridization the samples were placed on a magnetic separator to concentrate the beads and the supernatant carefully removed. The beads were then immediately re-suspended in 40 µl of 2×SSPE at 50°C and 5 ul of 5 µM of each detector oligonucleotide. The beads were then held at 50°C for a further 30 min with gentle shaking.

After this secondary hybridization/wash the beads were magnetically concentrated on a magnetic separator and washed twice with 100 ul of 2×SSPE at 50°C. The beads were then re-suspended in 40 µl 1×TMAC buffer (5 M tetramethilamonium chloride, 20% sarkosyl, 1 M Tris HCL pH 8) at 50°C along with 12 µl reporter mix (10 µg/ml streptavidin phycoerythrin conjugate in TMAC). Labeling was allowed to continue for 5 min in the MAGPIX instrument at 50°C then the signals were measured and the data exported to Excel 2010 (Microsoft, Seattle, USA).

In order to resolve unexpected results we PCR amplified and cycle sequenced a portion of the 16S rRNA gene which contained the region targeted in our assay, using primers 16SF-5′GRGRTACTCGAGTGGCGAAC3′ and 16SR-5′GACGACGGGTAGCCGGCC.

## Results

**Figure 2:**
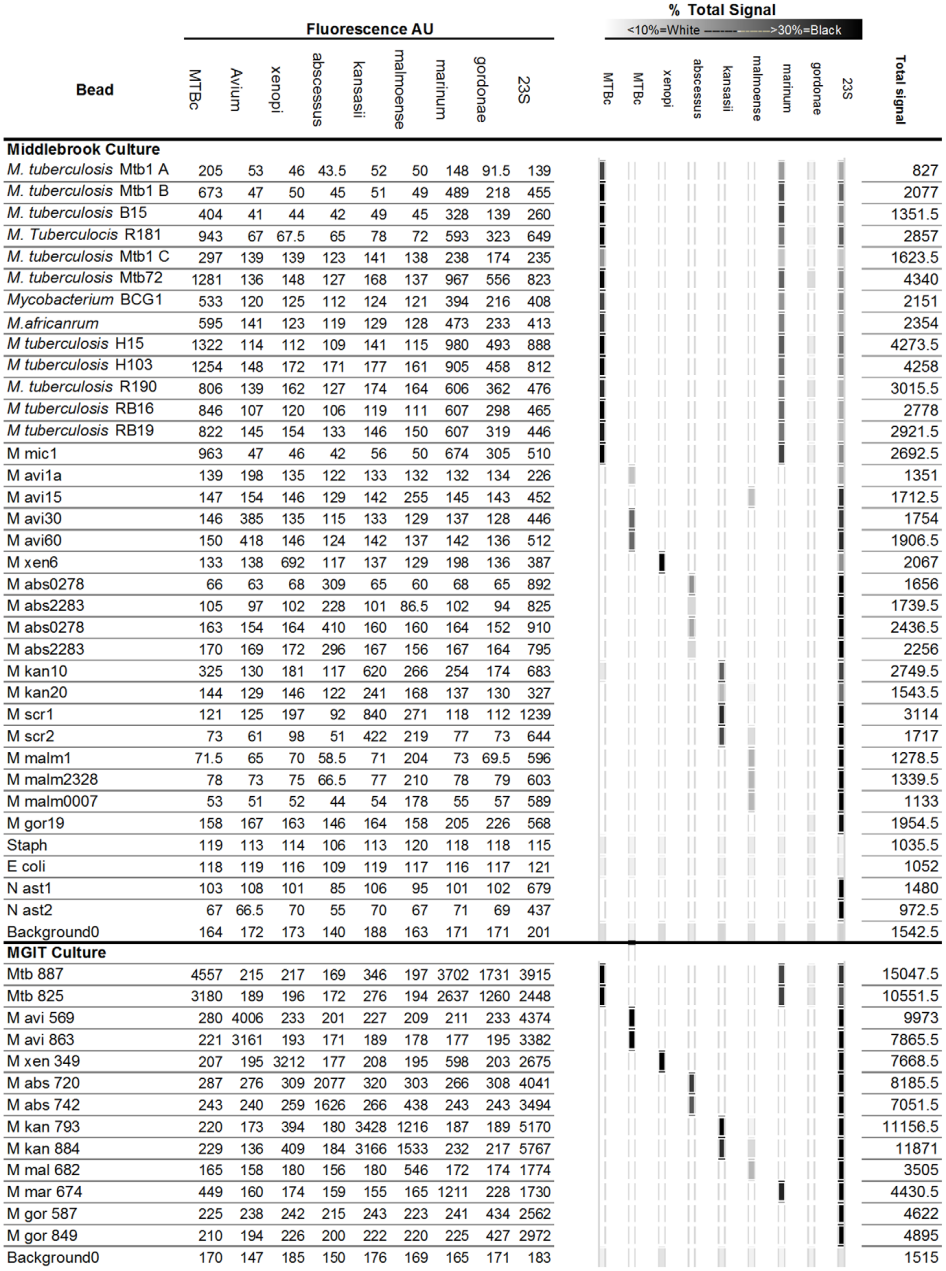
Results of hybridiations of, 31 Middlebrook cultures, 13 MGIT cultures, and 4 controls, to the 9 bead array. Fluorescence intensity in arbitrary units is indicated on the left hand side as determined by the MAGPIX machine, this data is visualized in the form of a “line probe assay” (Excel 2010, Microsoft, Seattle, USA) on the right hand side where the % of the total signal in each assay resulting from each specific bead is indicated as a grey scale (where <10% of total signal from a bead species is white, and >30% of total signal from a bead species is black).

The rCapA designed gave a characteristic profile for members of the MTB complex which was easily recognized and independent of the signal strength; total signals in arbitrary units (AU) ranged from 827 to 15,047 resulting in a characteristic profile in each case (Figure 2). During all our experiments the MTB complex probe invariably gave a signal for the MTBC species tested that was at least as high as the 23S positive control bead. In all other species tested the signal from the MTB complex probe was either absent or dramatically lower (always less than half) the signal obtained from the 23S positive control (Figure 2). All members of the MTB complex tested also demonstrated some cross reaction with the *M. marinum* probe but this did not result in misidentification as the *M. marinum* strain tested gave only a weak signal with the MTB complex probe (Figure 2).

**Figure 3:**
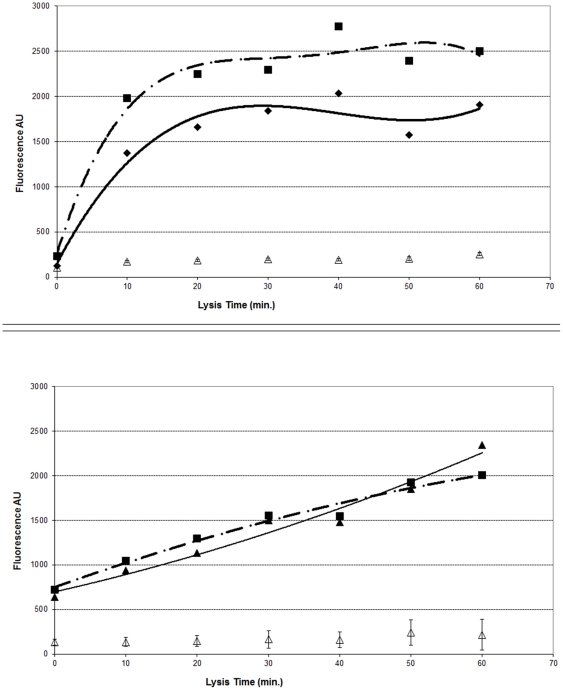
Effect of increasing lysis time on the yield of rRNA from an NTM (*M. avium*) and an *M. tuberculosis* culture. Solid lines and filled triangles/diamonds indicate the species specific bead signal, dashed lines and filled squares represent the 23S rRNA control signal, empty triangles indicate the average signal from the beads targeting other species (error bars +− one standard deviation). Lysis was performed at 30 Hz in the presence of Zirconium beads for pulses of 10 minutes. Aliquots of the prepared mycobacteria were removed after each period of shaking collected and analysed together in the rRNA capture MAGPIX assay. Upper graph *M. avium* lower graph *M. tuberculosis*.

**Figure 4:**
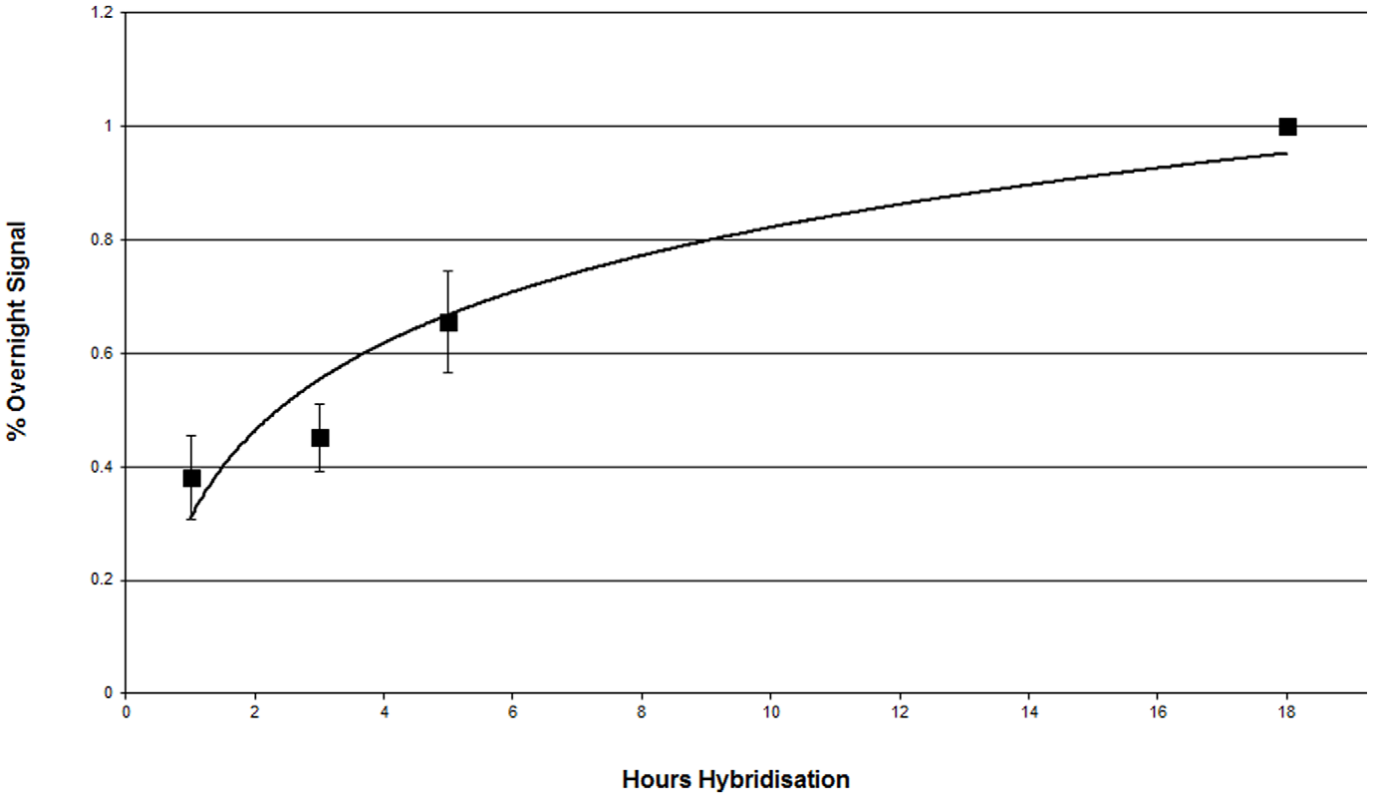
Effect of hybridisation time on the signal obtained for 3 independent experiments, error bars indicate +− one standard deviation.

Effective lysis was essential to obtain good signal and complete lysis of the MTBC strains tested was not achieved using our lysis protocol (Figure 3). Reduced hybridisation time also resulted in a lower signal with 50% of the overnight signal obtained after between four and six hours (Figure 4).

Background negative control samples and unrelated genera tested, *Staphylococcus aureus* and *Escherichia coli*, gave no detectable signal with the array. We also tested two mycolic acid containing, GC rich bacteria, identified based on partial 16S rRNA sequence as *Nocadia asteroides* and *Gordonia bronchialis*, which were originally obtained from sputum culture. These strains gave no detectable signal with the 16S rRNA probes, but yielded a clear signal with the 23S positive control bead (Figure 2).

One culture initially identified as *M. avium* (*M. avium* 15) gave a signal with the *M. malmoense* beads and the 23S positive control but no signal with the other beads including the *M. avium* probe. Because of this unexplained result we amplified and sequenced the respective part of the 16S rRNA of this strain and confirmed it had been misidentified in our collection [17]. Thus the *M. avium, M. xenopi, M. abscessus, M. kansasii*, and *M. marinum* probes exclusively reacted with members of their respective species (Figure 2). The *M. kansasii* probe also reacted with the two *M. scrofulaceum* isolates tested (Mscr1 and Mscr2, Figure 2). The *M. gordonae* probe we selected was unfortunately not functional (Figure 2).

## Discussion

The recent availability of a robust and relatively cost effective reader for a 50 plex liquid bead array (Luminex MAGPIX) stimulated us to explore the possibility of using this system to identify *Mycobacterium* cultures based on a method previously applied to the identification of other species [10] and mRNA expression analysis in Staphylococci [11].We developed an eight bead array to capture and discriminate 16S rRNA from members of the *Mycobacterium* genus and a ninth bead to nonspecifically capture 23S rRNA from mycobacteria and related genera to act as a positive control for the presence of GC rich gram variable organisms.

The samples from MGIT cultures tested gave higher signals (Figure 2, lower section), in part presumably as these samples were more highly concentrated during processing (see methods) but also possibly due to the more rapid growth of the mycobacteria in this medium and associated increase in rRNA expression. As we did not have access to automated culture in our laboratory, these samples were not tested directly but transported to our laboratory after they were flagged as positive by the machine. Future work should include testing in a more realistic “diagnostic setting”.

Our current rCapA is functional and reveals that in principle this is a highly efficient and robust approach for the identification of mycobacteria. However, there is still considerable potential left to improve the performance of the assay. Most striking are the experiments on the effect of lysis time on the signal obtained. For the experiments reported in Figure 3, lysis by shaking was allowed to continue for 10 min at 30 Hz. Increasing the shaking time for a *M. avium* culture demonstrated lysis of this species was almost complete after 10 min (Figure 3, upper graph) and optimal lysis was achieved after approximately 20 min of shaking (2×10 min with a 5 min pause). Further shaking did not result in an increase in the signal but, importantly, also no decrease in signal. Thus, there is no evidence that prolonged shaking resulted in damage or loss of rRNA (Figure 3A). In contrast for an *M. tuberculosis* culture prolonged shaking resulted in a steady almost linear increase in the signal obtained (Figure 3, lower graph). This indicates that the lysis achieved for MTB complex cultures with our current protocol was incomplete and a more efficient lysis system would result in increased signal/sensitivity. Alternative lysis methods should be explored; other workers have for instance developed simple disposable lysis chambers [18], [19] which would appear to be ideal for this type of assay.

We typically allowed hybridizations to continue overnight and analysed the results the next morning. It was also possible to perform the assay in one day, with a 4–5 hour hybridization step (Figure 4). Under these conditions approximately 60% of the overnight signal was obtained. The total signal obtained did not alter the interpretation of profiles obtained. For example, the first and second samples in Figure 2, *M. tuberculosis* Mtb1A and *M. tuberculosis* Mtb1B were hybridized for 4 hrs and 18 hrs, respectively.

A rule based and/or an automated species identification system is desirable and from these results appears realistic but a larger number of samples would need to be tested to develop and validate such an algorithm.

The *M. kansasii* probe also reacted with the two *M. scrofulaceum* isolates tested (Mscr1 and Mscr2, Figure 2), this was the result of an identical 16S rRNA sequence for the region targeted by the probe selected for this species. In order to discriminate these species a second region of the rRNA would need to be targeted. The *M. gordonae* probe we selected was unfortunately not functional (Figure 2) and 15 *M. gordonae* cultures tested all reacted only with the 23S positive control (results not shown). Thus, in common with most molecular assays not all probes designed *in silico* function in this assay even if the sequence is correct and probes must be experimentally screened for functionality.

Bacterial speciation is required when liquid culture is performed for tuberculosis diagnostics [2]. The rCapA presented here is particularly appealing for this purpose as unlike other “macro array” methods widely used in tuberculosis diagnostics [20] there is no need for a nucleic acid amplification/labelling step and associated need for stringent amplicon contamination controls. Combined with the recently developed robust liquid array readers we believe an expanded version of our array could considerably simplify the speciation of positive Mycobacterial cultures.

As a next step, extension and optimization of the panel of beads/probes and testing the performance on a larger collection of *Mycobacterium* isolates associated with disease and colonization or contamination should be undertaken. The analysis system used in this study is in principle capable of detecting 50 unique bead species although systems with even higher multiplexing capacity are available [21]. Our experiments also demonstrated that further optimization of the protocol can certainly streamline and increase the sensitivity of the rCapA. We regard it as unlikely that the sensitivity could be increased sufficiently to reliably directly detect *Mycobacterium* species from clinical material using the bead array detection system, although, other detection methods are being developed and it is not totally inconceivable that the rapid developments in DNA and nano technology relating to detection and signal amplification may ultimately be able to achieve direct identification of bacteria in clinical material using this approach. These developments in molecular detection [22], [23] deserve serious consideration by the microbial diagnostics community as and when they are reported.

The use of an amplification free system is potentially very robust utilizing mainly stable chemical reagents and is intrinsically resistant to amplicon contamination problems that have complicated the provision of amplification assays for infectious disease diagnostics where they are most needed. Finally, even moderate increases in sensitivity would allow RNA species other than the very highly expressed rRNA to be targeted e.g. highly expressed mRNA species such as rpoB mRNA [11], [24] and thereby greatly extend the application of the rCapA by providing the potential to discriminate more closely related genetic clades, with identical 16S rRNA sequences and detect additional characteristics such as drug resistance.
